# Predicting Axial Length From Choroidal Thickness on Optical Coherence Tomography Images With Machine Learning Based Algorithms

**DOI:** 10.3389/fmed.2022.850284

**Published:** 2022-06-28

**Authors:** Hao-Chun Lu, Hsin-Yi Chen, Chien-Jung Huang, Pao-Hsien Chu, Lung-Sheng Wu, Chia-Ying Tsai

**Affiliations:** ^1^Graduate Institute of Business and Management, Chang Gung University, Taoyuan, Taiwan; ^2^Division of Cardiology, Department of Internal Medicine, Chang Gung Memorial Hospital, Taoyuan, Taiwan; ^3^Department of Ophthalmology, Fu Jen Catholic University Hospital, New Taipei City, Taiwan; ^4^School of Medicine, College of Medicine, Fu Jen Catholic University, New Taipei City, Taiwan; ^5^Division of Cardiology, Department of Internal Medicine, Chang Gung Memorial Hospital, Chang Gung University College of Medicine, Taipei, Taiwan

**Keywords:** high myopia, choroidal thickness, axial length, machine learning, ensemble learning, optical coherence tomography (OCT)

## Abstract

**Purpose:**

We formulated and tested ensemble learning models to classify axial length (AXL) from choroidal thickness (CT) as indicated on fovea-centered, 2D single optical coherence tomography (OCT) images.

**Design:**

Retrospective cross-sectional study.

**Participants:**

We analyzed 710 OCT images from 355 eyes of 188 patients. Each eye had 2 OCT images.

**Methods:**

The CT was estimated from 3 points of each image. We used five machine-learning base algorithms to construct the classifiers. This study trained and validated the models to classify the AXLs eyes based on binary (AXL < or > 26 mm) and multiclass (AXL < 22 mm, between 22 and 26 mm, and > 26 mm) classifications.

**Results:**

No features were redundant or duplicated after an analysis using Pearson’s correlation coefficient, LASSO-Pattern search algorithm, and variance inflation factors. Among the positions, CT at the nasal side had the highest correlation with AXL followed by the central area. In binary classification, our classifiers obtained high accuracy, as indicated by accuracy, recall, positive predictive value (PPV), negative predictive value (NPV), F1 score, and area under ROC curve (AUC) values of 94.37, 100, 90.91, 100, 86.67, and 95.61%, respectively. In multiclass classification, our classifiers were also highly accurate, as indicated by accuracy, weighted recall, weighted PPV, weighted NPV, weighted F1 score, and macro AUC of 88.73, 88.73, 91.21, 85.83, 87.42, and 93.42%, respectively.

**Conclusions:**

Our binary and multiclass classifiers classify AXL well from CT, as indicated on OCT images. We demonstrated the effectiveness of the proposed classifiers and provided an assistance tool for physicians.

## Introduction

Myopia is a common disease among Asian people, and its incidence has increased worldwide. Holden et al. (1) estimated that the global prevalence of myopia would reach 49.8% in 2050 along with 9.8% for high myopia, and the myopia rate in East Asia would increase from 51.6 to 65.3%, the highest in the world, in the next 3 decades. Among Taiwanese schoolchildren evaluated between 1983 and 2017, the myopia rate quintupled from 5.37 to 25.41% for 7-year-olds and more than doubled (from 30.66 to 76.67%) for 12-year-olds (2).

Eyes with a spherical equivalence (SE) of less than -6.00 D were defined as having high myopia, and high myopia is correlated with axial length longer than 26.0 mm (3, 4). High myopia is associated with increased risks of cataract, glaucoma, retinal detachment, and maculopathy (5). These ocular complications of high myopia become more common with advanced age and may eventually lead to blindness (5, 6). Morgan et al. (6) suggested that the elongated AXL is the underlying mechanism of myopia development and progression. Choroid, located at the exterior of the retina and which provides blood supply to the outer portion of the retina, has been reported to be thinner in myopic than emmetropic eyes and is related to AXL elongation (7). Choroid thinning not only correlates with myopia progression but is also related to other complications, such as staphyloma and chorioretinal atrophy in high myopia (7–14). In addition to longer AXL, CT is also lower in older adults and in women (15).

Artificial intelligence (AI) is being used in medicine. In ophthalmology, color fundus images are commonly used for machine training in disease diagnosis, such as for diabetic retinopathy (DR), (16, 17) age-related macular disease (AMD), (18, 19) and glaucoma (20). Asaoka et al. (21) classified open-angle glaucoma and healthy eyes using deep learning algorithm trained on color fundus images from 159 patients (including 51 with glaucoma). Hemelings et al. identified pathologic myopia from color fundus images by means of Convolutional Neural Network (CNN) (22). Optical coherence tomography (OCT) has become one of the most effective imaging modalities in the diagnosis of various retinal conditions by providing high-resolution, cross-sectional images of the entire retina and choroid (23). The long wavelength (870 nm) used for scanning in spectral-domain OCT (SD-OCT) enables better penetration and ensures high-resolution retina and choroid images. Machine learning and deep learning have been successfully applied in OCT images for biomarker identification in AMD (24). Since myopia is a rising problem in ophthalmology, OCT images have been used for AI prediction in myopic eyes recently (25–27).

In this study, we focused on the relationship between CT and AXL. Since OCT is a common exam in clinics for patients with retinal diseases, glaucoma, and cataract surgery, it is meaningful to access more information from the existed exam images. With SD-OCT images from eyes with different refraction status and AXL, we investigated the utility of machine learning algorithms for predicting AXL and proposed a multiclass classifier of AXL by means of the CTs (28). In this study, five machine learning base algorithms [3 layers backpropagation neural network (BPN), support vector machine (SVM), random forest (RF), adaptive boosting (AdaBoost), extreme gradient boosting (XGBoost)] are used to construct classifiers for binary and multiclass classifications. The proposed classifiers can quickly and accurately predict the axial length by means of the choroid thickness (CT) and help us to understand the contribution of choroidal change in the etiology of myopia.

## Materials and Methods

This retrospective cohort study adhered to the tenets of the Declaration of Helsinki. This study was approved by the Institutional Review Board of Fu Jen Catholic University Hospital (FJUH).

## Data Sets

### Participants

Patients with OCT image findings taken from and who underwent AXL evaluation in CY Tsai’s and CJ Huang’s clinics in the ophthalmology department at FJUH at any period from Sep. 2017 to Dec. 2019 were included in this study. We collected comprehensive information for participants’ sex, age, body height, body weight, and best-corrected visual acuity (29, 30). Patients with incomplete data or retinopathies, such as diabetic retinopathy, age-related macular degeneration, and history of previous photodynamic therapy, were excluded from the study.

#### Optical Coherence Tomography Machine and Scanning Settings

**FIGURE 1:**
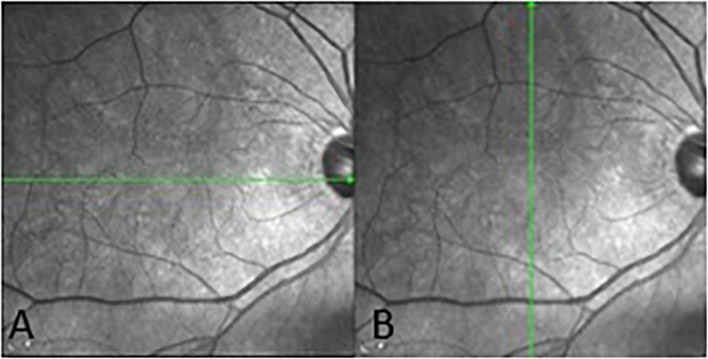
Cross-sectional **(A)** and longitudinal **(B)** choroidal images from SD-OCT.

**FIGURE 2:**
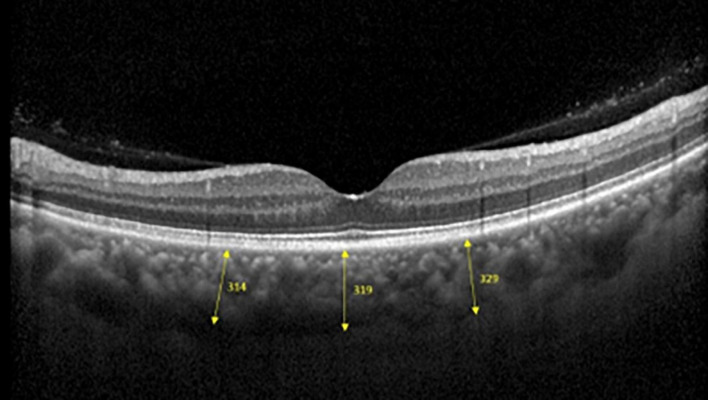
Three positions at which choroid thicknesses was indicated in OCT images.

Spectralis SD-OCT equipment (Heidelberg Engineering, Heidelberg, Germany) was used to evaluate CT in both eyes; OCT was performed in the daytime. Cross-sectional and longitudinal scanning was performed in each eye (Figure 1). The SD-OCT uses a super luminescence diode with an average wavelength of 870 nm as a light source, an 8-um axial resolution, and a 10-μm transverse resolution in tissue. The position of fovea was defined as the anatomical depression of macula. The CT was measured at 6 points: central fovea, 3 mm nasal, and 3 mm temporal to the fovea at cross-sectional image and central fovea, 3 mm superior, and 3 mm inferior to the fovea at longitudinal image (Figure 2). Each image was measured by 2 investigators independently and rechecked by a third investigator.

#### AXL Measurement

The AXL of the eyes was evaluated by a non-contact technique by using a Lenstar LS 900 platform (HAAG-Streit, Mason, OH, United States).

#### Features

**FIGURE 3:**
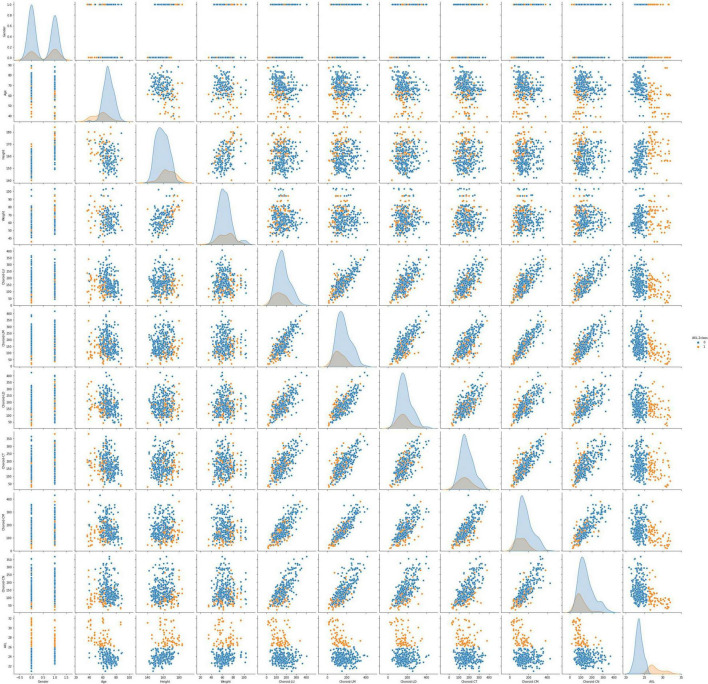
Pairwise scatter plots of all features with binary classification.

**FIGURE 4:**
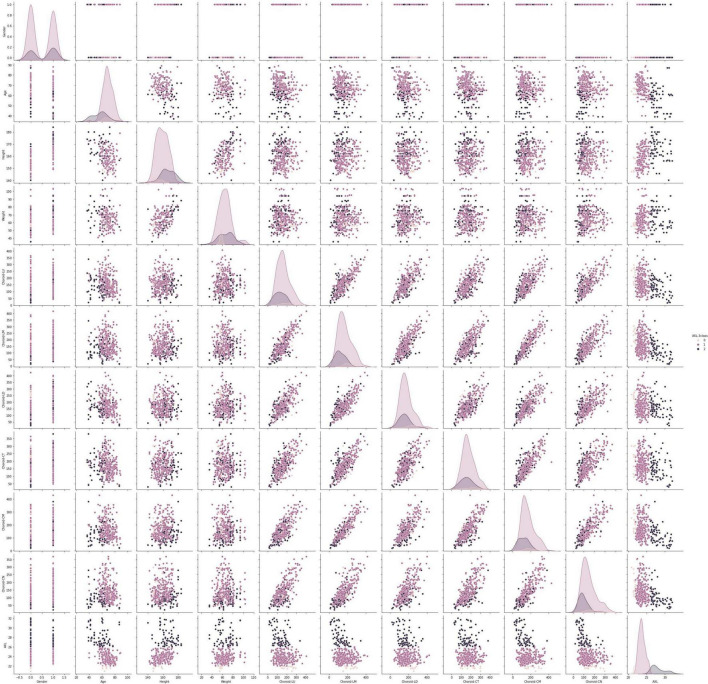
Pairwise scatter plots of all features with multiclass classification.

This data set had 11 features (Table 1), which were (1) participants’ gender, age, height, and weight; (2) 3 cross-sectional CTs; (3) 3 longitudinal CTs; and (4) AXL. The pairwise scatter plots of all features with binary and multiclass classifications are shown in Figures 3, 4, respectively. Figures 3, 4 clearly show that the relationships of all pairs of two features are almost non-linear.

**TABLE 1:** Features in this study.

No.	Feature name	Description	Data type
1.	Gender	0 for male and 1 for female.	Nominal
2.	Age	The age of subject.	Continuous
3.	Height	The height of subject (cm).	Continuous
4.	Weight	The weight of subject (kg).	Continuous
5.	Choroid-LU	Up thicknesses of longitudinal choroid.	Continuous
6.	Choroid-LM	Middle thicknesses of longitudinal choroid.	Continuous
7.	Choroid-LD	Down thicknesses of longitudinal choroid.	Continuous
8.	Choroid-CT	Temporal thicknesses of cross sections choroid.	Continuous
9.	Choroid-CM	Middle thicknesses of cross sections choroid.	Continuous
10.	Choroid-CN	Nasal thicknesses of cross sections choroid.	Continuous
11.	AXL	Axial length of eyes	Continuous

#### Binary and Multiclass Classification of Axial Length

This study trained and validated the classifiers, which predicted the class of AXL of each eye using binary and multiclass classifications. In binary classification, we classified eyes into AXL < 26 mm and AXL> 26 mm; in multiclass classification, we classified eyes into AXL < 22 mm, 22 mm> AXL < 26 mm, and AXL > 26 mm (31–34).

### Development of Classifiers by Machine Learning Algorithms

#### Algorithm Selection

In this study, we analyzed 710 OCT images from 355 eyes of 188 patients. However, 710 images are quite low for a CNN algorithm. An appropriate number of samples depends on the specific problem, and it should be tested for each case individually. But a rough rule of thumb is to train a CNN algorithm with a data set larger than 5,000 samples for effective generalization of the problem. Our previous study used data augmentation to increase this study’s image samples and utilized a CNN algorithm to construct the image classifier through OCT images. However, the image classifier obtains a low accuracy. For obtaining satisfactory results, this research does not use simple algorithms to construct the linear classifier and selects state-of-the-art or strong algorithms to construct the non-linear classifier. Therefore, the selected algorithms are BNN, SVM, RF, AdaBoost, and XGBoost. RF, AdaBoost, and XGBoost are also the ensemble learning.

Essentially, ensemble learning algorithms feature the combination of several weak classifiers to form a strong one with bagging or boosting approaches. The bagging approach trains many individual models in a parallel way, and each model is trained by a random subset of the data. Boosting approach trains a bunch of individual models in a sequential manner, and each individual model learns from mistakes made by the previous model. The Ensemble learning algorithms obtain less bias, less variance, and better results than traditional machine learning in general. Friedman et al. (35) indicated that boosting approach results in dramatic performance improvements and no additional requirements for the dataset and classifiers.

The RF, AdaBoost, and XGBoost are based on the bagging, boosting, and hybrid bagging and boosting approaches. AdaBoost, one of the first boosting algorithms adapted to solve practical problems, uses multiple iterations to create a strong learner by iteratively adding weak learners. Gradient boosting, a generalization of AdaBoost, is one of the most powerful techniques for building predictive models. The main objective of gradient boosting is to minimize the loss function by adding weak learners using a gradient descent algorithm. XGBoost is an extension of gradient-boosted decision trees and has the following advantages: regularized learning, gradient tree boosting, and shrinkage with column subsampling. Since the used ensemble learning algorithms in this study always have the hyperparameter—*n_estimators* (the number of estimators), the *n_estimators* means the number of the individual model will be performed. Therefore, ensemble learning algorithms always spend much more time than BNN and SVM calculation time.

#### Classifiers Construction Process

**FIGURE 5:**
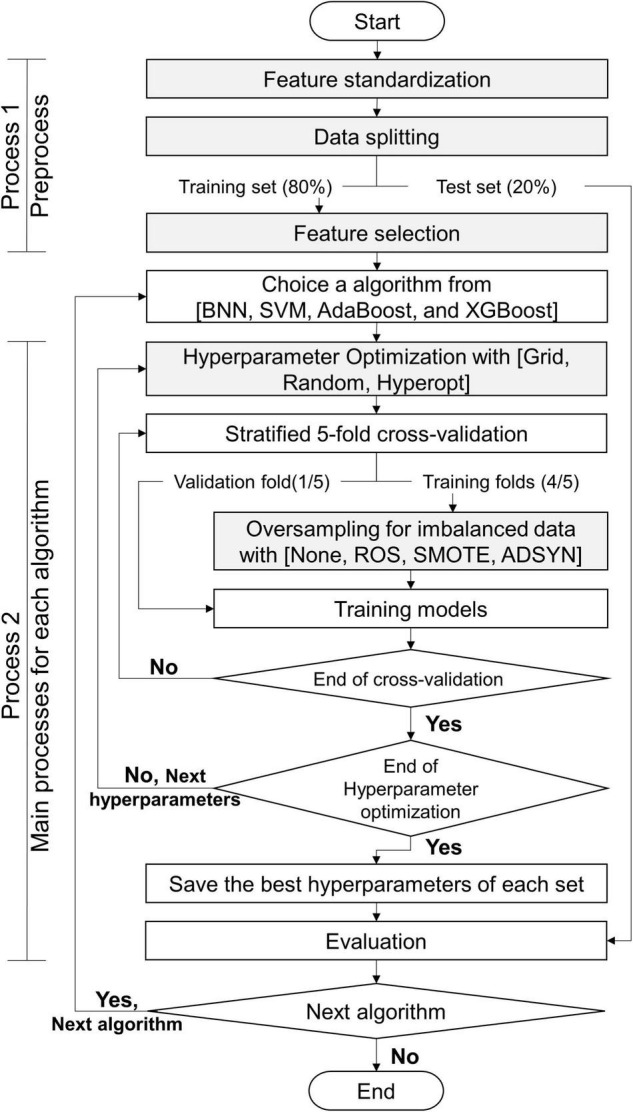
Flowchart of this study.

The processes of this study are exhibited in Figure 5: process 1 (preprocess) and process 2 (primary processes for each algorithm). Before we constructed the classifiers, the data set was preprocessed by using process 1. This study utilized 5 algorithms (BNN, SVM, RF, AdaBoost, and XGBoost) to predict myopia by means of the CTs. We constructed 2 classifiers for binary and multiclass classifications for each algorithm. Without loss of generality, all models constructed by each algorithm were executed by process 2. Finally, this study obtained the appropriate features, suggesting resample methods, and the appropriate values of hyperparameters for each algorithm with the target classifications. The details of gray steps exhibited in Figure 5 are described in subsections *feature standardization*, *data splitting*, *feature selection*, *hyperparameter Optimization*, and *Oversampling of Imbalanced Data*.

#### Feature Standardization

To reduce the training phase’s processing time, we standardized numerical features by removing their means and scaling to unit variance through the formula as follows.

Feature with normalization = (feature – feature’s mean)/feature’s standard deviation.

#### Data Splitting

The 355 tuples in this imbalanced data were collected from 188 patients with 355 eyes. Each tuple represented a completely eyeball’s six choroidal thicknesses based on cross-sectional and longitudinal scanning images. Table 4 indicates that the proportion of AXL < 26 mm to AXL > 26 mm was 0.7944:0.2056 in binary classification. In multiclass classification, the proportion of AXL < 22 mm, between 22 and 26 mm, and > 26 mm was 0.0394:0.7549:0.2056. We split the imbalanced data into training and test sets based on a uniform random distribution, and the percentage ratio of training and test sets followed 80 and 20% with patient level (no patient across both training and test sets), where each set shared a similar proportion of all categories. The training set was used in *feature selection*, *hyperparameter optimization*, and o*versampling*. Finally, the test set was used to evaluate all metrics of each set {algorithm, hyperparameters search method, oversampling method} in Evaluation step of process 2 in Figure 2 and the comparisons of AXL class prediction between humans and classifiers.

#### Feature Selection

This study used 3 methods: Pearson’s correlation coefficient (Pearson), variance inflation factor (VIF), and least absolute shrinkage and selection operator (Lasso) to evaluate and select the appropriate features based on the training set. In the training set, one tuple only contained one eye’s features. Therefore, the feature selection can purely evaluate the relationships of the features within one eye. The three feature selection methods were performed sequentially independently, and any feature detected as redundant or useless by any method will be removed in this step.

Pearson’s *r* indicated the linear relationship between a given feature and class label. The VIF measured how substantially the variance of an independent feature was influenced by other independent features. If the VIF of the target feature was > 10, we eliminated the target feature. Lasso performs covariate selection by forcing the sum of the absolute value of the regression coefficients to be less than a fixed value, which forced certain coefficients to be 0. The order of importance of input features made the fitted model more interpretable. LASSO utilized the L1 penalty to select the most feature at once based on a given lambda value. Compared with LASSO, Elastic net used a penalty mixed L1 and L2 norms, and Elastic net is hard to obtain the clear order of importance input features.

Finally, 100 different lambda values (1.00, 0.99,…, 0.01) with descending order were used in LASSO, and we only kept the lambda values that LASSO selection an additional new significant feature in S Table 5.

#### Hyperparameter Optimization

Each algorithm had its hyperparameters that need to be tuned because the appropriate hyperparameters were very different for the algorithm applied in a different dataset. That is, different hyperparameters will most influence the performances of an algorithm. The search scopes for consideration of hyperparameters for all algorithms in this study (BNN, SVM, RF, AdaBoost, XGBoost) were listed in Supplementary Appendix-I. Since the combinations of hyperparameters for each algorithm were numerous, this study used three search methods [grid search, random search, and Hyperopt search proposed by Bergstra et al. (36)] to search the appropriate hyperparameters. Each search method searched and evaluated the possible hyperparameters among the scopes in Supplementary Appendix-I, and the search method obtained the best hyperparameters of the algorithm with the target training set by the main matric—F1-score. Finally, each algorithm will pick the best hyperparameters among the three search methods.

Grid search searched the performances with all combinations of hyperparameters with the specific fixed values in their scopes. Random and Hyperopt searches selected the hyperparameters’ with the possible real numbers from the specific intervals; therefore, random search and Hyperopt search selected more floating values for the hyperparameters that were not listed in the grid search. The numbers of grid search and random search were the same. The speed of Hyperopt search was much slower than grid search and random search because Hyperopt analyzes and improves the values of hyperparameters after each iteration; therefore, the search number of Hyperopt was 1/2 that of grid search. In Supplementary Appendix-I, the search numbers of {grid search, random search, and Hyperopt} for BNN, SVM, RF, AdaBoost, and XGBoost are {162, 162, 81}, {20,100, 20,100, 10,050}, {15,000, 15,000, 7,500}, {24,000, 24,000, 12,000}, and {25,920, 25,920, 12,960}, respectively. Therefore, there were 212,955 searches for binary classification, and the total number of searches in this study is 425,910 for both binary and multiclass classifications.

For each search in the three search methods, stratified fivefold cross-validation (CV) was used to evaluate the performance of the current hyperparameters. Although the fivefold CV will take a 5-times validation time than the holdout method, the trained model will not be easy to overfit for a specific validation set and reduce the bias and variance of the performance estimate.

#### Oversampling for Imbalanced Data

For each split of fivefold CV, we oversampled the training fold to avoid the imbalanced issue. The use of this technique increased the number of samples of the smaller-sized categories for the sample sizes to be consistent among all categories. The oversampled samples of smaller-sized categorized of the training fold appeared only in the training fold. This study used three oversampling techniques as follows.

Random oversampling (ROS): Randomly sample the tuples in the categories of smaller sample sizes.

Synthetic minority oversampling technique (SMOTE): For the categories of smaller sample sizes, find a sample *x* and its *k*-nearest neighbor samples *x*_j_(*j* = 1,…,*k*). Select one individual $x_{\text{j}}^{\prime }$ from *x*_j_ and create a new sample based on the linear combination of *x*_i_ and $x_{\text{j}}^{\prime }$.

Adaptive synthetic sampling (ADASYN): ADASYN is a technique based on the SMOTE algorithm for generating synthetic data. The difference between ADASYN and SMOTE is that ADASYN implements a methodology that detects those samples of the minority class found in spaces dominated by the majority class to generate samples in the lower density areas of the minority class. ADASYN focuses on those samples of the minority class that are difficult to classify because they are in a low-density area.

Finally, the numbers of training and validation folds with the three oversampling techniques and original data set are presented in Table 2.

**TABLE 2:** Size of original data sets and oversampled data sets.

Oversampling Set		None			ROS/SMOTE/ ADASYN		
			
		Training (80%)	Test (20%)	Total	Training	Test	Total
Binary	AXL < 26 mm	226	56	282	226	56	282
	AXL > 26 mm	58	15	73	226	15	241
	Sum	284	71	355	452	71	523
Multiclass	AXL < 22 mm	11	3	14	215	3	218
	22 mm>AXL < 26 mm	215	53	268	215	53	268
	AXL> 26 mm	58	15	73	215	15	230
	Sum	284	71	355	645	71	716

### Algorithm Evaluation and Statistical Analysis

#### Evaluating Metrics

Because the data set in this study was imbalanced and had many classes, accuracy alone was not sufficient to indicate the classifiers’ performance. Therefore, accuracy, recall (sensitivity), PPV (precision), NPV, F1 score, Specificity, and AUC were used for binary classification. For multiclass classification, this study used accuracy, weighted recall (sensitivity), weighted PPV, weighted NPV, weighted F1 score, weighted Specificity, and macro AUC where macro AUC is the macro average of multiple one-vs-rest AUCs. Five algorithms (BPN, SVM, AdaBoost, XGBoost, RF), three hyperparameter optimizations (Grid Search, Random Search, Hyperopt), and four oversampling techniques (None, ROS, SMOTE, and ADASYN) were used to construct classifiers with training set based on the best hyperparameters obtained in Hyperparameter Optimization for binary and multiclass classifications. The numbers of training and test sets with the three oversampling techniques and original data set are presented in Table 2. In total, 120 experiment results with test set are reported for binary and multiclass classification. The details of the experiment results of binary and multiclass classifications are listed in Supplementary Appendix-II,III respectively.

### AXL Prediction

For evaluation of AXL prediction from human eyes, 2 ophthalmologists and two medical students were asked to predict AXL of the eyes by the 10 features (age, sex, height, weight, choroidal thickness from the 6 points). They were blind to the AXL during prediction, and the results were checked and compared with the results from AI algorithm with *t*-test by another person in our group.

## Results

### Demographics

In this study, 710 OCT images of 355 eyes (172 left and 183 right) of 188 patients were collected. All patients had complete sex, age, body height, and body weight data in their medical records. In total, 87 (46.28%) men and 101 (53.72%) women participated. The average age was 66.49 ± 9.73 years, average body height was 159.8 ± 8.72 cm, and average body weight was 64.8 ± 12.1 kg (Table 3). The average AXL was 24.55 ± 2.26 mm at the right eye and 24.61 ± 2.21 mm at the left eye. The average CT at the central fovea, 3 mm nasal, and 3 mm temporal to the fovea on cross-sectional image was 161.46 ± 75.17 mm, 137.88 ± 69.29 mm, and 170.01 ± 65.56 mm at the right eye, respectively; 162.48 ± 72.16 mm, 139.58 ± 67.30 mm, 175.86 ± 66.50 mm at the left eye, respectively. The average CTs at the central fovea, 3 mm superior, and 3 mm inferior to the fovea on longitudinal image were 161.56 ± 74.9, 172.67 ± 71.56, and 158.16 ± 66.01 mm at the right eye, respectively; 162.64 ± 71.03, 161.24 ± 69.22, and 175.32 ± 62.97 mm at the left eye, respectively. In our binary classification, there were 282 (79.44%) eyes with AXL < 26 mm and 73 (20.56%) eyes with AXL ≥ 26 mm; in the multiclass classification, there were 14 (3.94%) eyes with AXL < 22 mm, 268 (75.49%) between 22 and 26 mm, and 73 (20.56%) eyes with AXL ≥ 26 mm (Table 4).

**TABLE 3:** Characteristics of participants.

Feature	Number (%)	Feature	Number (%)
Gender		Height (cm, mean = 159.8, SD = 8.72)	
Male	87 (46.3%)	<150	24 (12.8%)
Female	101 (53.7%)	150–159.9	67 (35.6%)
Age (mean = 66.5, SD = 9.73)		160–169.9	65 (34.6%)
<40	3 (1.6%)	170–179.9	29 (15.4%)
40–49	10 (5.3%)	>179.9	3 (1.6%)
50–59	19 (10.1%)	Weight (kg, mean = 64.8, SD = 12.1)	
60–69	85 (45.2%)	<50	14 (7.4%)
70–79	58 (30.9%)	50–59.9	54 (28.7%)
>79	13 (6.9%)	60–69.9	55 (29.3%)
		70–79.9	45 (23.9%)
		80–89.9	12 (6.4%)
		>89.9	8 (4.3%)

**TABLE 4:** Class label criteria in terms of AXL.

	Binary classification	
Class	Rule	Number (%)
0	AXL < 26 mm	282 (79.44%)
1	AXL ≥ 26 mm	73 (20.56%)
Class	Multiclass Classification Rule	Number (%)
0	AXL < 22 mm	14 (3.94%)
1	22 mm ≤ AXL < 26 mm	268 (75.49%)
2	AXL ≥ 26 mm	73 (20.56%)

**TABLE 5:** Superior performance in binary classification.

Classifier	Algorithm	Hyper. Opt.	Over sampling	Accuracy	Recall	PPV	NPV	F1-score	Specificity	AUC
1	SVM	Random	ROS	92.96%	100%	73.68%	100%	84.85%	91.22%	95.61%
2	AdaBoost	Random	ADASYN	94.37%	92.86%	81.25%	98.18%	86.67%	94.73%	93.80%
3	AdaBoost	Hyperopt	ROS	92.30%	71.43%	90.91%	93.33%	80.00%	98.25%	84.84%

### Results of Pearson, VIF, and LASSO

The Pearson results of all eyes revealed that the 10 features were substantially correlated with the AXL (all *P* < 0.05). Age had the strongest correlation, followed by height, Choroid-CN, Choroid-LM, Choroid-CM, Choroid-LU, Choroid-LD, Choroid-CT, gender, and weight. Height and weight had positive correlations with AXL, and men had longer AXL, but the other features had negative correlation with AXL (Supplementary Tables 1, 2). Regarding the CT between the left and right eye, Choroid-CM and Choroid-CN had symmetric properties between the left and right eye because their *r* coefficients were higher than others. However, the others were asymmetric (Supplementary Table 3). The results of VIF revealed that the 10 features exhibited no multicollinearity because no feature’s VIF was > 10 (Supplementary Table 4).

The sequence of the features’ coefficient becoming non-zero under Lasso with decreasing α is listed as follows: age, Choroid-CN, Choroid-LM, Height, Choroid-CM, Choroid-LU, weight, Choroid-CT, Choroid-LD, and gender. Height and male gender are positively correlated with AXL. Among the 6 CTs, Choroid-CT and Choroid-LD features were positively correlated to AXL, compared with other features. However, based on the Lasso with α = 0.01, the coefficients of gender, choroid-LD, and weight were relatively small (Supplementary Table 5). After the Pearson, VIF, and Lasso analyses were conducted, all of the 10 features were found to be non-redundant. Therefore, all 10 of the features were used for constructing the classifiers.

### Results of Proposed Classifiers

Classifier 3 obtained the best PPV and Specificity of 90.91 and 98.25%. Classifier 4 obtains the best PPV and Specificity of 92.21 and 93.37%. Tables 6, 7 list some classifiers with the best metrics for binary and multiclass classifications, respectively. All metrics in Tables 6, 7 are calculated from an independent test set described in the Data Splitting. Classifier 1 (SVM with random search and ROS oversampling), Classifier 2 (AdaBoost with random search and ADASYN oversampling), and Classifier 3 (AdaBoost with Hyperopt search and ROS oversampling) have different best metrics for binary classification. Classifier 1 obtains the best recall, NPV, and AUC of 100, 100, and 95.61%, respectively. Classifier 2 obtains the best accuracy and F1-score of 94.37 and 86.67%. Classifier 3 obtained the best PPV and Specificity of 90.91 and 98.25%.

**TABLE 6:** Superior performances in multiclass classification.

Classifier	Algorithm	Hyper. Opt.	Over sampling	Accuracy	Recall (weighted)	PPV (weighted)	NPV (weighted)	F1-score (weighted)	Specificity (weighted)	AUC (macro)
4	SVM	Random	SMOTE	78.87%	78,87%	92.21%	62.56%	83.17%	93.37%	88.71%
5	AdaBoost	Grid	ROS	88.73%	88.73%	86.16%	82.28%	87.43%	74.75%	93.06%
6	XGBoost	Grid	SMOTE	85.92%	85.92%	83.06%	78.89%	84.32%	65.07%	93.42%
7	XGBoost	Random	ROS	87.32%	87.32%	84.96%	85.83%	85.78%	68.27%	84.64%
										

**TABLE 7:** The comparison and test results in binary classification.

	Item	Accuracy	Recall	PPV	NPV	F1-score	Specificity	AUC
Student 1	Metric	80.28%	66.67%	52.63%	90.38%	58.82%	83.93%	75.30%
	*p*-value	0.006**	0.000***	0.000***	0.023*	0.000***	0.019*	0.001**
Student 2	Metric	56.34%	6.67%	5.56%	73.58%	6.06%	69.64%	38.15%
	*p*-value	0.000***	0.000***	0.000***	0.000***	0.000***	0.000***	0.000 ***
OPH 1	Metric	77.46%	60.00%	47.37%	88.46%	52.94%	82.14%	71.07%
	*p*-value	0.002**	0.000***	0.000***	0.010*	0.000***	0.010*	0.000***
OPH 2	Metric	80.28%	60.00%	52.94%	88.89%	56.25%	85.71%	72.86%
	*p*-value	0.006**	0.000***	0.000***	0.012*	0.000***	0.035*	0.000***

p value: * < 0.05, ** < 0.01, *** < 0.001.

For multiclass classification, Classifier 4 (SVM with grid random and SMOTE oversampling), Classifier 5 (AdaBoost with grid search and ROS oversampling), Classifier 6 (XGBoost with grid search and SMOTE oversampling), and Classifier 7 (XGBoost with random search and ROS oversampling) have different best metrics. Classifier 4 obtains the best PPV and Specificity of 92.21 and 93.37%. Classifier 5 obtains the best accuracy, weighted recall, and weighted F1 score of 88.73, 88.73, and 87.43%, respectively. Classifier 6 and Classifier 7 obtain the best macro AUC (93.51%) and weighted NPC, respectively.

Among all metrics, F1-score is the main metric in this study because F1-score seeks the balance of Recall and PPV for the imbalanced dataset. In clinical application, it can help doctors utilize the ensemble learning with the balance of positive prediction and effective medical resource use. However, assessing a model with the best F1-score and poor other metrics is inappropriate. It is still very important to comprehensively consider all metrics. Based on Tables 6, 7, the proposed Classifiers 2 and 5 are excellent models for detecting myopia with binary and multiclass classifications. It is possible to classify AXL > or < 26 mm by CTs with the proposed Classifier 2 because Classifier 2 has no low performances of all metrics. AXL < 22 mm, between 22 and 26 mm, ≥ 26 mm can be classified based on CTs with the proposed Classifier 5 because Classifier 5 has good performances for all metrics.

Based on Table 6, Classifier 1 (SVM) has the best recall, NPV, and AUC but a poor PPV. Classifier 2 (AdaBoost) obtains the best accuracy and F1-score, and it also has the second-best recall, PPV, NPV, F1-score, and specificity. Additional, the gaps of recall, NPV, and AUC between Classifier 1 and Classifier 2 are small. Because Classifier 2 is more stable than Classifiers 1 and 3 and has no low performances of all metrics, Classifier 2 is recommended to classify AXL > or < 26 mm by CTs.

Based on Table 7, Classifier 4 (SVM) has the best the best PPV (weighted) and specificity (weighted) but very poor accuracy, recall (weighted), and NPV (weighted). Classifier 6 (XGBoost) and Classifier 7 (XGBoost) respectively, have the best AUC (macro) and NPV (weighted) but medium remaining metrics. Classifier 5 (AdaBoost) obtains the best Accuracy, recall, and F1-score, and it also has the second-best PPV (weighted), NPV (weighted), specificity (weighted), and AUC (macro). Because Classifier 5 is excellent and stable than other three Classifiers (4, 6, and 7), Classifier 5 can be used to classify AXL < 22 mm, AXL between 22 and 26 mm, and AXL ≥ 26 mm by CTs.

The appropriate values of hyperparameters of classifiers in Tables 5, 6 are obtained by Hyperparameter Optimization, and the details of values of hyperparameters are listed in Supplementary Appendix-IV.

### The AXL Prediction

To compare the results of AXL prediction based on the 10 features between the proposed classifiers and ophthalmologists, we recruited 2 ophthalmologists and 2 medical students to predict AXL in binary and multiclass classification based on the same 10 features. In the results, the accuracy was 48.61–69.44%, PPV (weighted) was 61.50–76.08%, Recall (weighted) was 48.61–69.44%, F1 score (weighted) was 54.29–71.92%, NPV (weighted) was 38.77–53.71%, and AUC (macro) was 49.07–63.03%. The results were considerably less accurate than those from our developed classifiers in Tables 5, 6.

### The Comparisons of AXL Class Prediction Between Humans and Classifiers

To compare the AXL class prediction between the proposed classifiers and ophthalmologists, we recruited two ophthalmologists (OPHs) and two medical students to compare binary and multiclass classification based on the same test set with ten features (without AXL feature). Since the test set size is over 30, this study used the test of proportion to verify the performance of results. The null and alternative hypotheses are *H*_0_:*p*_Human_≥*p*_AI_ and *H*_1_:*p*_Human_ < *p*_AI_, where *p*_*Human*_ and *p*_*AI*_ are the metrics of human performances and proposed classifiers, respectively. The comparison and test results are listed in Tables 7, 8.

**TABLE 8:** The comparison and test results in multiclass classification.

	Item	Accuracy	Recall (weighted)	PPV (weighted)	NPV (weighted)	F1-score (weighted)	Specificity (weighted)	AUC (macro)
Student 1	Metric	67.61%	67.61%	70.60%	41.54%	64.92%	36.98%	52.29%
	*p*-value	0.001**	0.001**	0.012*	0.000***	0.001**	0.000***	0.000***
Student 2	Metric	47.89%	47.89%	60.86%	38.91%	53.59%	50.25%	49.07%
	*p*-value	0.000***	0.000***	0.000***	0.000***	0.000***	0.001**	0.000***
OPH 1	Metric	53.52%	53.52%	63.76%	41.16%	57.21%	53.00%	53.26%
	*p*-value	0.000***	0.000***	0.001**	0.000***	0.000***	0.003**	0.000***
OPH 2	Metric	66.20%	66.20%	72.85%	50.61%	69.04%	59.85%	63.03%
	*p*-value	0.001**	0.001**	0.025*	0.000 ***	0.004**	0.029*	0.000***

p value: * < 0.05, ** < 0.01, *** < 0.001.

In Tables 7, 8, the human performances’ results of accuracy, recall, PPV, NPV, F1-score, specificity, and AUC, respectively, are 47.89–80.28%, 6.67–67.61%, 5.56–72.85%, 38.91–90.38%, 6.06–69.04%, 36.98–85.71%, and 38.15–75.30%. Compared with the same metrics of proposed classifiers 2 and 5 in Tables 6, 7, all tests of proportion rejected H0. It demonstrated that the proposed classifiers outperform the human performances.

## Discussion

In this study, we proposed that Classifiers 1–6 can predict AXL by means of patients’ age, sex, height, weight, and CT measured from OCT images. Studies have reported that CT is negatively correlated with AXL, and people with high myopia tend to have a thinner choroid (7–11). However, few studies have assessed the prediction of AXL by means of CT. In the proposed classifiers, the binary prediction has accuracy, recall, PPV > 90%, and NPV > 85%; multiclass prediction has accuracy, recall, PPV, and NPV > 80%, which is substantially better than prediction by ophthalmologists in this study. The 10 selected features were correlated with AXL, and the correlation was confirmed by the Pearson, VIF, and Lasso analyses. In the Pearson and Lasso analyses, age had the highest negative correlation with AXL. This observation may result from the difference in prevalence of myopia among various age groups. Studies have reported that from 1983 to 2017, the prevalence of myopia in the same age group substantially increased in Taiwan, (2) which leads to more incidence of myopic eyes in younger patients in our cohort. Another possible reason for this correlation is that whereas older patients could have various conditions that require ophthalmic clinic follow-up, younger patients seldom have severe eye disease that requires clinic visits and image studies, except for those patients with myopia or high myopia who were at risk of retinal complications. Among the six choroid locations, CT at the nasal side in cross-section (Choroid-CN) was the thinnest. This result is compatible with those of El-Shalzly et.al. (9) and Gupta et al. (13) which have demonstrated that CT at the nasal side was thinner in patients with myopia and emmetropia. Furthermore, CT at the nasal side also has the highest negative correlation with AXL both in Pearson and Lasso analyses. Although the exact mechanism requires further investigation, this result demonstrated that CT at the nasal side is essential for AXL prediction and possibly essential in myopia development.

Regarding myopia prediction, Varadarajan et al. (37) developed a model to predict SE from color fundus images. Shi et al. used CNN to predict myopia with absolute mean error of 1.115 D in SE from a color fundus image (38). We chose AXL classifications as our prediction. Our patients’ average age was ≈ 66 years, and most of our participants had various severities of cataract; many of the participants had received cataract surgery. Because SE may be influenced by lens condition and cylinder, which are not directly related to retinal or choroidal condition, we considered AXL as a more accurate feature to reflect a patient’s myopic condition. Because some of our patients had retinal disease, such as AMD, DME, retinoschisis, or myopic CNV, the retinal condition may vary between patients and even among multiple visits for the same patient. Thus, we recorded the CT to avoid the potential variation of retinal condition. Dong et al. (38) predicted AXL and subfoveal CT from a color fundus image with high accuracy (39). In their heat map analysis, they demonstrated that different areas of the macula on the fundus image were used to predict various AXL. The entire macular region, foveal region, and extrafoveal region were used to predict AXL < 22 mm, from 22 to 26 mm, and > 26 mm, respectively. In our study, we measured the CT at fovea and perifovea, and predicted AXL from the features. Among all of the CTs at various positions, the nasal side had the highest correlation with AXL, followed by the central part, and the result was unanimous in binary and multiclass classifications. Compatible with that of Dong et al. our results also demonstrated that the CT at the fovea and perifoveal region can predict AXL in various classifications.

In classifier construction, feature scaling is an essential preprocessing step in AI. Before one evaluates and selects the features, all features must be standardized to prevent redundancy or duplication. We used Pearson, VIF, and Lasso analyses to select the proper features. Pearson’s *r* indicates the linear relationship between a given feature and class label. The *p* value indicates the probability that a feature is uncorrelated with the class label, per the method of Kowalski (40). The VIF measures how substantially the variance of an independent feature is influenced by other independent features. If the VIF of the target feature was > 10, we eliminated the target feature. The Lasso method was proposed by Santosa and Symes (41) and popularized by Tibshirani (42). Lasso performs covariate selection by forcing the sum of the absolute value of the regression coefficients to be less than a fixed value, which forces certain coefficients to be 0.

In hyperparameter optimization, Random Search is more effective than Grid Search for a fixed search number; (36) Hyperopt obtains superior values of hyperparameters within the same executing time (43). Our data set was imbalanced both in binary and multiclass classifications, and the use of such imbalanced data to train the model may yield a biased result. The method we used, oversampling, is a popular technique for treating imbalanced data to avoid the aforementioned problems.

The ensemble-learning approach has also been used in ophthalmology to diagnose DR and interpret OCT imaging (44, 45). In our study, we used 5 algorithms (BNN, SVM, RF, AdaBoost, and XGBoost) to construct the classifiers of axial length through CTs. The classifiers constructed by ensemble approach (RF, AdaBoost, and XGBoost) outperformed those constructed by single machine learning approach (BNN and SVM). Those constructed by AdaBoost (Classifiers 1, 2, 4, and 5) and XGBoost (Classifiers 3 and 6) had the most optimal performance. Essentially, AdaBoost and XGBoost features the combination of several weak classifiers to form a strong one with a boosting approach. For the 2 algorithms (AdaBoost and XGBoost), the boosting approach plays a crucial role in dealing with the bias-variance tradeoff, and the boosting approach is considered more effective.

In this study, we successfully conducted AXL classification at the accuracies of 94.34 and 88.73% for binary and multiclass classifications by hyperparameter optimization, oversampling, and boosting algorithms. The high prediction accuracy in our binary and multiclass classification could be attributed to two main reasons. First, all of our imputed CT were repeated measured and rechecked by an ophthalmologist familiar with OCT images to ensure the accuracy of each measurement and avoid segmentation errors. Second, the seven final classifiers were chosen from 8,518,200 candidates [from 425,910 searches, each went through 20 (4 oversampling and five cross-validation) complete experiments], thus enabled our model to have high accuracies.

This study has several limitations. First, our sample size was relatively small, especially those with AXL < 22 mm or > 26 mm; the distribution of AXLs is also relatively imbalanced. Second, the process of collecting the 10 features was time consuming. Considerable time and effort were required to measure the thickness of the choroid of 6 positions from 2 OCT images of each eye and to collect data on sex, age, weight, and height of each of the participants. Among the features we recorded, AXL did not increase with age after adulthood, and weight may change without change of AXL; these potentially induced bias in our results. Third, the manual measurement of CT may cause bias or inconsistency. Future studies should address these limitations, and we expect to conduct more investigations using a larger data set on the classification and diagnosis of eye diseases which may be revealed by SD-OCT.

We demonstrated the effectiveness of the proposed classifiers in classification prediction from medical data and provided an assistance tool for physicians.

## Data Availability Statement

The raw data supporting the conclusions of this article will be made available by the authors, without undue reservation.

## Ethics Statement

The studies involving human participants were reviewed and approved by Fu Jen Catholic University Hospital, Fu Jen Catholic University. Written informed consent for participation was not required for this study in accordance with the national legislation and the institutional requirements.

## Author Contributions

CY-T and H-CL: conceptualization and writing – original preparation. H-CL: methodology and software. C-YT, H-YC, and C-JH: data collection. H-CL and P-HC: formal analysis. C-YT, H-YC, L-SW, and P-HC: writing – review and edition. C-YT: supervision. H-CL, L-SW, and P-HC: funding acquisition. All authors have read and agreed to the published version of the manuscript.

## Conflict of Interest

The authors declare that the research was conducted in the absence of any commercial or financial relationships that could be construed as a potential conflict of interest.

## Publisher’s Note

All claims expressed in this article are solely those of the authors and do not necessarily represent those of their affiliated organizations, or those of the publisher, the editors and the reviewers. Any product that may be evaluated in this article, or claim that may be made by its manufacturer, is not guaranteed or endorsed by the publisher.
