# Supplementary material for: Predicting Axial Length From Choroidal Thickness on Optical Coherence Tomography Images With Machine Learning Based Algorithms
Source: Front Med (Lausanne). 2022 Jun 28;9:850284. doi: 10.3389/fmed.2022.850284 (PMC9273745; doi:10.3389/fmed.2022.850284)
Supplement: Supplementary file 1 [file Data_Sheet_1.docx]

Supplement Table 1. The Pearson correlation results of all eyes

|  | Gender | Age | Height | Weight | Choroid-LU | Choroid-LM | Choroid-LD | Choroid-CT | Choroid-CM | Choroid-CN | Y AXL |
| --- | --- | --- | --- | --- | --- | --- | --- | --- | --- | --- | --- |
| Gender | N/A | -0.0276 | 0.6834 | 0.3882 | 0.0682 | 0.1724 | 0.1232 | 0.1168 | 0.1536 | 0.1647 | 0.1266 |
| Age | -0.0276 | N/A | -0.3106 | -0.1193 | -0.1707 | -0.0509 | -0.2105 | -0.1773 | -0.0823 | -0.0177 | -0.5199 |
| Height | 0.6834 | -0.3106 | N/A | 0.5155 | 0.0559 | 0.0892 | 0.0913 | 0.0777 | 0.0838 | 0.0774 | 0.3625 |
| Weight | 0.3882 | -0.1193 | 0.5155 | N/A | 0.0164 | 0.0433 | 0.0719 | -0.0167 | 0.0108 | 0.0357 | 0.1056 |
| Choroid-LU | 0.0682 | -0.1707 | 0.0559 | 0.0164 | N/A | 0.7909 | 0.7409 | 0.7123 | 0.7729 | 0.7525 | -0.2569 |
| Choroid-LM | 0.1724 | -0.0509 | 0.0892 | 0.0433 | 0.7909 | N/A | 0.7857 | 0.7307 | 0.8897 | 0.7755 | -0.3272 |
| Choroid-LD | 0.1232 | -0.2105 | 0.0913 | 0.0719 | 0.7409 | 0.7857 | N/A | 0.7146 | 0.7345 | 0.6721 | -0.1694 |
| Choroid-CT | 0.1168 | -0.1773 | 0.0777 | -0.0167 | 0.7123 | 0.7307 | 0.7146 | N/A | 0.7827 | 0.6911 | -0.1487 |
| Choroid-CM | 0.1536 | -0.0823 | 0.0838 | 0.0108 | 0.7729 | 0.8897 | 0.7345 | 0.7827 | N/A | 0.8089 | -0.3089 |
| Choroid-CN | 0.1647 | -0.0177 | 0.0774 | 0.0357 | 0.7525 | 0.7755 | 0.6721 | 0.6911 | 0.8089 | N/A | -0.3443 |
| Y AXL | 0.1266 | -0.5199 | 0.3625 | 0.1056 | -0.2569 | -0.3272 | -0.1694 | -0.1487 | -0.3089 | -0.3443 | N/A |

Supplement Table 2. The Pearson p-value results of all eyes

|  | Gender | Age | Height | Weight | Choroid-LU | Choroid-LM | Choroid-LD | Choroid-CT | Choroid-CM | Choroid-CN | Y AXL |
| --- | --- | --- | --- | --- | --- | --- | --- | --- | --- | --- | --- |
| Gender | N/A | 0.6036 | **0** | **0** | 0.1995 | **0.0011** | **0.0201** | **0.0276** | **0.0037** | **0.0018** | **0.0169** |
| Age | 0.6036 | N/A | **0** | **0.0243** | **0.0012** | 0.3386 | **0.0001** | **0.0008** | 0.121 | 0.7389 | **0** |
| Height | **0** | **0** | N/A | **0** | 0.293 | 0.093 | 0.0852 | 0.1436 | 0.1145 | 0.1452 | **0** |
| Weight | **0** | **0.0243** | **0** | N/A | 0.7577 | 0.4156 | 0.1761 | 0.7532 | 0.8389 | 0.502 | **0.0464** |
| Choroid-LU | 0.1995 | **0.0012** | 0.293 | 0.7577 | N/A | **0** | **0** | **0** | **0** | **0** | **0** |
| Choroid-LM | **0.0011** | 0.3386 | 0.093 | 0.4156 | **0** | N/A | **0** | **0** | **0** | **0** | **0** |
| Choroid-LD | **0.0201** | **0.0001** | 0.0852 | 0.1761 | **0** | **0** | N/A | **0** | **0** | **0** | **0.0013** |
| Choroid-CT | **0.0276** | **0.0008** | 0.1436 | 0.7532 | **0** | **0** | **0** | N/A | **0** | **0** | **0.0049** |
| Choroid-CM | **0.0037** | 0.121 | 0.1145 | 0.8389 | **0** | **0** | **0** | **0** | N/A | **0** | **0** |
| Choroid-CN | **0.0018** | 0.7389 | 0.1452 | 0.502 | **0** | **0** | **0** | **0** | **0** | N/A | **0** |
| Y AXL | **0.0169** | **0** | **0** | **0.0464** | **0** | **0** | **0.0013** | **0.0049** | **0** | **0** | N/A |

Supplement Table 3. Pearson correlation result between left and right eyes

|  |  | Right eye | | | | | |
| --- | --- | --- | --- | --- | --- | --- | --- |
|  |  | Choroid-LU | Choroid-LM | Choroid-LD | Choroid-CT | Choroid-CM | Choroid-CN |
| Left eye | Choroid-LU | 0.6804 | 0.6496 | 0.639 | 0.63 | 0.6652 | 0.6669 |
|  | Choroid-LM | 0.6465 | 0.7217 | 0.6551 | 0.591 | 0.7134 | 0.6654 |
|  | Choroid-LD | 0.6002 | 0.6346 | 0.6394 | 0.5369 | 0.5994 | 0.5565 |
|  | Choroid-CT | 0.5954 | 0.6104 | 0.6382 | 0.6345 | 0.6345 | 0.5617 |
|  | Choroid-CM | 0.6846 | 0.7387 | 0.6801 | 0.6423 | **0.7548** | 0.6908 |
|  | Choroid-CN | 0.672 | 0.6903 | 0.6191 | 0.5836 | 0.71 | **0.7317** |

Supplement Table 4. VIF results of features 1-10

| Feature | VIF |
| --- | --- |
| Gender | 2.1094 |
| Age | 1.3644 |
| Height | 2.581 |
| Weight | 1.3946 |
| Choroid-LU | 3.5962 |
| Choroid-LM | 6.5057 |
| Choroid-LD | 3.2792 |
| Choroid-CT | 3.0593 |
| Choroid-CM | 6.6431 |
| Choroid-CN | 3.4504 |

Supplement Table 5. LASSO results with certain features

| α | Gender | Age | Height | Weight | Choroid-LU | Choroid-LM | Choroid-LD | Choroid-CT | Choroid-CM | Choroid-CN |
| --- | --- | --- | --- | --- | --- | --- | --- | --- | --- | --- |
| 0.76 | 0.0 | **-0.38** | 0.0 | 0.0 | 0.0 | 0.0 | 0.0 | 0.0 | 0.0 | 0.0 |
| 0.67 | 0.0 | -0.47 | 0.0 | 0.0 | 0.0 | 0.0 | 0.0 | 0.0 | 0.0 | **-0.09** |
| 0.65 | 0.0 | -0.49 | 0.0 | 0.0 | 0.0 | **-0.01** | 0.0 | 0.0 | 0.0 | -0.10 |
| 0.31 | 0.0 | -0.75 | **0.29** | 0.0 | 0.0 | -0.25 | 0.0 | 0.0 | 0.0 | -0.29 |
| 0.25 | 0.0 | -0.80 | 0.34 | 0.0 | 0.0 | -0.27 | 0.0 | 0.0 | **-0.03** | -0.31 |
| 0.1 | 0.0 | -0.94 | 0.47 | 0.0 | **-0.1** | -0.29 | 0.0 | 0.0 | -0.09 | -0.34 |
| 0.08 | 0.0 | -0.95 | 0.50 | **-0.03** | -0.11 | -0.29 | 0.0 | 0.0 | -0.1 | -0.34 |
| 0.03 | 0.0 | -0.97 | 0.59 | -0.13 | -0.19 | -0.29 | 0.0 | **0.21** | -0.24 | -0.37 |
| 0.01 | **0.03** | -0.98 | 0.63 | -0.19 | -0.25 | -0.35 | **0.10** | 0.33 | -0.32 | -0.39 |
