# Supplementary material for: Predicting Axial Length From Choroidal Thickness on Optical Coherence Tomography Images With Machine Learning Based Algorithms
Source: Front Med (Lausanne). 2022 Jun 28;9:850284. doi: 10.3389/fmed.2022.850284 (PMC9273745; doi:10.3389/fmed.2022.850284)
Supplement: Supplementary file 2 [file Data_Sheet_2.docx]

APPENDIX-I. The details of experiment results of binary classification

- Values and ranges of hyperparameters for BNN

| Hyperparameter | Grid | Random and Auto |
| --- | --- | --- |
| Number of nodes in 1^st^ hidden layer | 10 | 10-20 |
| Number of nodes in 2^nd^ hidden layer | 10-20 (step 2) | 10-20 (step 1) |
| Activation function in 1^st^ hidden layer | {tanh, sigmoid, relu} | |
| Activation function in 2^nd^ hidden layer | {tanh, sigmoid, relu} | |
| Optimizers | {Adam, Adadelta, Adagrad} | |
| Number of searches | Grid, Random: 162. Auto: 81 | |

- Values and ranges of hyperparameters for SVM

| Hyperparameter | Grid | Random and Auto |
| --- | --- | --- |
| Kernel function | {linear, poly, rbf, sigmoid} | {linear, poly,rbf, sigmoid} |
| Value of degree (for poly) | 1-5 (step 1) | 1-5 (step 1) |
| Value of coef0 (for poly, sigmoid) | 0-2 (step 0.2) | [0-2] |
| Value of L2 regularization | {0.01,2^-5^,2^-4^,2^-3^,2^-2^,2^-1^,0.1, 1,5,10,15,20,25,40,50} | [0.01-50] |
| Value of gamma | {0.01,2^-5^,2^-4^,2^-3^,2^-2^,2^-1^,1, 1.5, 2,2.5,3,3.5,4,‘scale’,‘auto’} | [0.01-4] |
| Number of searches | Grid, Random: 20100. Auto: 10050 | |

- Values and ranges of hyperparameters for RF

| Hyperparameter | Grid | Random and Auto |
| --- | --- | --- |
| Measures of impurity | {gini, entropy} | |
| Number of trees | 100-500 (step 100) | [100-500] |
| Maximum depth of the tree | 2-20 (step 2) | [2-20] |
| Number of min samples split | 2-10 (step 2) | [2-10] |
| Number of min samples leaf | 1-9 (step 2) | [1-9] |
| Number of max features | {auto, log2, None} | |
| Bootstrap samples | {True, False} | |
| Number of searches | Grid, Random: 15000. Auto: 7500 | |

- Values and ranges of hyperparameters for AdaBoost

| Hyperparameter | Grid | Random and Auto |
| --- | --- | --- |
| Criterion of decision tree | {gini, entropy} | |
| Maximum depth of decision tree | 2-10 (step 2) | [2-10] |
| Max features of decision tree | {auto, log2, None} | |
| Min samples split of decision tree | 2-10 (step 2) | [2-10] |
| Min samples leaf of decision tree | 1-9 (step 2) | [1-9] |
| Maximum number of estimators | 50-350 (step 100) | [50-350] |
| Value of learning rate | {0.2, 0.1, 0.01, 0.001} | [0.2-0.01] |
| Algorithm | {SAMME, SAMME.R} | |
| Number of searches | Grid, Random: 24000. Auto: 12000 | |

- Values and ranges of hyperparameters for XGBoost

| Hyperparameter | Grid | Random and Auto |
| --- | --- | --- |
| Maximum depth of a tree | 2-10 (step 2) | [2-10] |
| Value of gamma | 0-10 (step 2) | [0-10] |
| Min sum of child weight rate | 0-10 (step 2) | [0-10] |
| Value of subsample ratio | 0.4-1.0 (step 0.2) | [0.4-1] |
| Columns' subsample ratio by tree | 0.6-1.0 (step 0.2) | [0.6-1.0] |
| Value of learning rate | {0.3, 0.1, 0.01, 0.001} | [0.001-0.3] |
| Maximum number of estimators | 100-300 (step 100) | [100-300] |
| Number of searches | Grid, Random: 25920. Auto: 12960 | |

APPENDIX-II. The details of experiment results of binary classification

| Algorithm | Hyper. Opt. | Over sampling | Accuracy | Recall | PPV | NPV | F1-score | Specificity | AUC |
| --- | --- | --- | --- | --- | --- | --- | --- | --- | --- |
| BPN | Grid | None | 0.845 | 0.467 | 0.700 | 0.869 | 0.560 | 0.946 | 0.707 |
|  |  | ROS | 0.859 | 0.933 | 0.609 | 0.979 | 0.737 | 0.839 | 0.886 |
|  |  | SMOTE | 0.789 | 0.929 | 0.481 | 0.977 | 0.634 | 0.754 | 0.841 |
|  |  | ADASYN | 0.817 | 0.867 | 0.542 | 0.957 | 0.667 | 0.804 | 0.835 |
|  | Random | None | 0.831 | 0.667 | 0.588 | 0.907 | 0.625 | 0.875 | 0.771 |
|  |  | ROS | 0.817 | 0.643 | 0.529 | 0.907 | 0.581 | 0.860 | 0.751 |
|  |  | SMOTE | 0.915 | 0.733 | 0.846 | 0.931 | 0.786 | 0.964 | 0.849 |
|  |  | ADASYN | 0.648 | 0.571 | 0.296 | 0.864 | 0.390 | 0.667 | 0.619 |
|  | Hyperopt | None | 0.915 | 0.714 | 0.833 | 0.932 | 0.769 | 0.965 | 0.840 |
|  |  | ROS | 0.803 | 0.867 | 0.520 | 0.957 | 0.650 | 0.786 | 0.826 |
|  |  | SMOTE | 0.859 | 0.857 | 0.600 | 0.961 | 0.706 | 0.860 | 0.858 |
|  |  | ADASYN | 0.577 | 0.933 | 0.326 | 0.964 | 0.483 | 0.482 | 0.708 |
| SVM | Grid | None | 0.873 | 1.000 | 0.609 | 1.000 | 0.757 | 0.842 | 0.921 |
|  |  | ROS | 0.887 | 0.867 | 0.684 | 0.962 | 0.765 | 0.893 | 0.880 |
|  |  | SMOTE | 0.930 | 0.800 | 0.857 | 0.947 | 0.828 | 0.964 | 0.882 |
|  |  | ADASYN | 0.901 | 0.867 | 0.722 | 0.962 | 0.788 | 0.911 | 0.889 |
|  | Random | None | 0.901 | 1.000 | 0.667 | 1.000 | 0.800 | 0.877 | 0.939 |
|  |  | ROS | 0.930 | 1.000 | 0.737 | 1.000 | 0.848 | 0.912 | 0.956 |
|  |  | SMOTE | 0.915 | 1.000 | 0.700 | 1.000 | 0.824 | 0.895 | 0.947 |
|  |  | ADASYN | 0.887 | 1.000 | 0.636 | 1.000 | 0.778 | 0.860 | 0.930 |
|  | Hyperopt | None | 0.915 | 0.733 | 0.846 | 0.931 | 0.786 | 0.964 | 0.849 |
|  |  | ROS | 0.859 | 1.000 | 0.583 | 1.000 | 0.737 | 0.825 | 0.912 |
|  |  | SMOTE | 0.915 | 1.000 | 0.700 | 1.000 | 0.824 | 0.895 | 0.947 |
|  |  | ADASYN | 0.873 | 1.000 | 0.609 | 1.000 | 0.757 | 0.842 | 0.921 |
| RF | Grid | None | 0.873 | 0.643 | 0.692 | 0.914 | 0.667 | 0.930 | 0.786 |
|  |  | ROS | 0.859 | 0.467 | 0.778 | 0.871 | 0.583 | 0.964 | 0.715 |
|  |  | SMOTE | 0.859 | 0.643 | 0.643 | 0.912 | 0.643 | 0.912 | 0.778 |
|  |  | ADASYN | 0.873 | 0.643 | 0.692 | 0.914 | 0.667 | 0.930 | 0.786 |
|  | Random | None | 0.789 | 0.733 | 0.500 | 0.918 | 0.595 | 0.804 | 0.768 |
|  |  | ROS | 0.859 | 0.643 | 0.643 | 0.912 | 0.643 | 0.912 | 0.778 |
|  |  | SMOTE | 0.887 | 0.714 | 0.714 | 0.930 | 0.714 | 0.930 | 0.822 |
|  |  | ADASYN | 0.845 | 0.643 | 0.600 | 0.911 | 0.621 | 0.895 | 0.769 |
|  | Hyperopt | None | 0.901 | 0.714 | 0.769 | 0.931 | 0.741 | 0.947 | 0.831 |
|  |  | ROS | 0.873 | 0.643 | 0.692 | 0.914 | 0.667 | 0.930 | 0.786 |
|  |  | SMOTE | 0.789 | 0.800 | 0.500 | 0.936 | 0.615 | 0.786 | 0.793 |
|  |  | ADASYN | 0.873 | 0.643 | 0.692 | 0.914 | 0.667 | 0.930 | 0.786 |
| AdaBoost | Grid | None | 0.887 | 0.571 | 0.800 | 0.902 | 0.667 | 0.965 | 0.768 |
|  |  | ROS | 0.915 | 0.786 | 0.786 | 0.947 | 0.786 | 0.947 | 0.867 |
|  |  | SMOTE | 0.887 | 0.786 | 0.688 | 0.945 | 0.733 | 0.912 | 0.849 |
|  |  | ADASYN | 0.915 | 0.714 | 0.833 | 0.932 | 0.769 | 0.965 | 0.840 |
|  | Random | None | 0.803 | 0.600 | 0.529 | 0.889 | 0.563 | 0.857 | 0.729 |
|  |  | ROS | 0.859 | 0.643 | 0.643 | 0.912 | 0.643 | 0.912 | 0.778 |
|  |  | SMOTE | 0.930 | 0.857 | 0.800 | 0.964 | 0.828 | 0.947 | 0.902 |
|  |  | ADASYN | 0.944 | 0.929 | 0.813 | 0.982 | 0.867 | 0.947 | 0.938 |
|  | Hyperopt | None | 0.887 | 0.643 | 0.750 | 0.915 | 0.692 | 0.947 | 0.795 |
|  |  | ROS | 0.930 | 0.714 | 0.909 | 0.933 | 0.800 | 0.982 | 0.848 |
|  |  | SMOTE | 0.887 | 0.714 | 0.714 | 0.930 | 0.714 | 0.930 | 0.822 |
|  |  | ADASYN | 0.930 | 0.857 | 0.800 | 0.964 | 0.828 | 0.947 | 0.902 |
| XGBoost | Grid | None | 0.831 | 0.800 | 0.571 | 0.940 | 0.667 | 0.839 | 0.820 |
|  |  | ROS | 0.803 | 0.800 | 0.522 | 0.938 | 0.632 | 0.804 | 0.802 |
|  |  | SMOTE | 0.859 | 0.929 | 0.591 | 0.980 | 0.722 | 0.842 | 0.885 |
|  |  | ADASYN | 0.887 | 0.857 | 0.667 | 0.962 | 0.750 | 0.895 | 0.876 |
|  | Random | None | 0.803 | 0.667 | 0.526 | 0.904 | 0.588 | 0.839 | 0.753 |
|  |  | ROS | 0.789 | 0.933 | 0.500 | 0.977 | 0.651 | 0.750 | 0.842 |
|  |  | SMOTE | 0.845 | 0.667 | 0.625 | 0.909 | 0.645 | 0.893 | 0.780 |
|  |  | ADASYN | 0.887 | 0.786 | 0.688 | 0.945 | 0.733 | 0.912 | 0.849 |
|  | Hyperopt | None | 0.887 | 0.600 | 0.818 | 0.900 | 0.692 | 0.964 | 0.782 |
|  |  | ROS | 0.915 | 0.786 | 0.786 | 0.947 | 0.786 | 0.947 | 0.867 |
|  |  | SMOTE | 0.930 | 0.929 | 0.765 | 0.981 | 0.839 | 0.930 | 0.929 |
|  |  | ADASYN | 0.873 | 0.667 | 0.714 | 0.912 | 0.690 | 0.929 | 0.798 |

APPENDIX-III. The details of experiment results of multiclass classification

| Algorithm | Hyper. Opt. | Over sampling | Accuracy | Recall (weighted) | Precision (weighted) | NPV (weighted) | F1-score (weighted) | Specificity (weighted) | AUC (macro) |
| --- | --- | --- | --- | --- | --- | --- | --- | --- | --- |
| BPN | Grid | None | 0.803 | 0.803 | 0.815 | 0.965 | 0.736 | 0.322 | 0.864 |
|  |  | ROS | 0.577 | 0.577 | 0.825 | 0.507 | 0.614 | 0.892 | 0.846 |
|  |  | SMOTE | 0.549 | 0.549 | 0.823 | 0.513 | 0.578 | 0.892 | 0.885 |
|  |  | ADASYN | 0.493 | 0.493 | 0.843 | 0.458 | 0.556 | 0.901 | 0.827 |
|  | Random | None | 0.859 | 0.859 | 0.859 | 0.747 | 0.859 | 0.747 | 0.909 |
|  |  | ROS | 0.437 | 0.437 | 0.775 | 0.479 | 0.425 | 0.840 | 0.784 |
|  |  | SMOTE | 0.493 | 0.493 | 0.717 | 0.451 | 0.559 | 0.714 | 0.642 |
|  |  | ADASYN | 0.493 | 0.493 | 0.767 | 0.466 | 0.511 | 0.818 | 0.831 |
|  | Hyperopt | None | 0.817 | 0.817 | 0.787 | 0.699 | 0.801 | 0.666 | 0.888 |
|  |  | ROS | 0.592 | 0.592 | 0.796 | 0.501 | 0.618 | 0.840 | 0.855 |
|  |  | SMOTE | 0.704 | 0.704 | 0.850 | 0.560 | 0.750 | 0.871 | 0.826 |
|  |  | ADASYN | 0.662 | 0.662 | 0.795 | 0.539 | 0.694 | 0.813 | 0.810 |
| SVM | Grid | None | 0.479 | 0.479 | 0.824 | 0.430 | 0.570 | 0.829 | 0.913 |
|  |  | ROS | 0.746 | 0.746 | 0.863 | 0.580 | 0.793 | 0.833 | 0.897 |
|  |  | SMOTE | 0.746 | 0.746 | 0.864 | 0.600 | 0.779 | 0.884 | 0.897 |
|  |  | ADASYN | 0.732 | 0.732 | 0.829 | 0.561 | 0.769 | 0.779 | 0.846 |
|  | Random | None | 0.775 | 0.775 | 0.891 | 0.606 | 0.814 | 0.885 | 0.906 |
|  |  | ROS | 0.761 | 0.761 | 0.890 | 0.600 | 0.798 | 0.924 | 0.875 |
|  |  | SMOTE | 0.789 | 0.789 | 0.912 | 0.626 | 0.832 | **0.934** | 0.887 |
|  |  | ADASYN | 0.845 | 0.845 | 0.873 | 0.707 | 0.858 | 0.794 | 0.892 |
|  | Hyperopt | None | 0.803 | 0.803 | 0.894 | 0.635 | 0.833 | 0.886 | 0.910 |
|  |  | ROS | 0.732 | 0.732 | 0.894 | 0.577 | 0.783 | 0.926 | 0.874 |
|  |  | SMOTE | 0.803 | 0.803 | 0.905 | 0.637 | 0.834 | 0.931 | 0.888 |
|  |  | ADASYN | 0.775 | 0.775 | 0.884 | 0.612 | 0.815 | 0.882 | 0.913 |
| RF | Grid | None | 0.789 | 0.789 | 0.788 | 0.684 | 0.760 | 0.458 | 0.908 |
|  |  | ROS | 0.831 | 0.831 | 0.808 | 0.843 | 0.796 | 0.464 | 0.909 |
|  |  | SMOTE | 0.845 | 0.845 | 0.816 | 0.805 | 0.822 | 0.557 | 0.892 |
|  |  | ADASYN | 0.859 | 0.859 | 0.831 | 0.825 | 0.839 | 0.606 | 0.906 |
|  | Random | None | 0.803 | 0.803 | 0.796 | 0.697 | 0.789 | 0.544 | 0.904 |
|  |  | ROS | 0.831 | 0.831 | 0.823 | 0.791 | 0.819 | 0.622 | 0.749 |
|  |  | SMOTE | 0.817 | 0.817 | 0.805 | 0.672 | 0.811 | 0.643 | 0.881 |
|  |  | ADASYN | 0.859 | 0.859 | 0.831 | 0.825 | 0.839 | 0.606 | 0.893 |
|  | Hyperopt | None | 0.859 | 0.859 | 0.831 | 0.789 | 0.843 | 0.651 | 0.904 |
|  |  | ROS | 0.845 | 0.845 | 0.815 | 0.768 | 0.827 | 0.602 | 0.910 |
|  |  | SMOTE | 0.817 | 0.817 | 0.784 | 0.695 | 0.798 | 0.550 | 0.906 |
|  |  | ADASYN | 0.887 | 0.887 | 0.860 | 0.857 | 0.872 | 0.703 | 0.907 |
| AdaBoost | Grid | None | 0.845 | 0.845 | 0.847 | 0.790 | 0.838 | 0.637 | 0.928 |
|  |  | ROS | 0.887 | 0.887 | 0.862 | 0.823 | 0.874 | 0.748 | 0.931 |
|  |  | SMOTE | 0.887 | 0.887 | 0.862 | 0.823 | 0.874 | 0.748 | 0.861 |
|  |  | ADASYN | 0.775 | 0.775 | 0.794 | 0.640 | 0.782 | 0.720 | 0.854 |
|  | Random | None | 0.803 | 0.803 | 0.804 | 0.718 | 0.781 | 0.503 | 0.911 |
|  |  | ROS | 0.831 | 0.831 | 0.821 | 0.764 | 0.823 | 0.656 | 0.916 |
|  |  | SMOTE | 0.803 | 0.803 | 0.805 | 0.687 | 0.803 | 0.690 | 0.887 |
|  |  | ADASYN | 0.831 | 0.831 | 0.831 | 0.732 | 0.831 | 0.732 | 0.926 |
|  | Hyperopt | None | 0.845 | 0.845 | 0.848 | 0.806 | 0.839 | 0.663 | 0.880 |
|  |  | ROS | 0.845 | 0.845 | 0.858 | 0.751 | 0.843 | 0.770 | 0.888 |
|  |  | SMOTE | 0.803 | 0.803 | 0.761 | 0.724 | 0.778 | 0.570 | 0.895 |
|  |  | ADASYN | 0.831 | 0.831 | 0.800 | 0.781 | 0.803 | 0.509 | 0.918 |
| XGBoost | Grid | None | 0.817 | 0.817 | 0.776 | 0.783 | 0.784 | 0.501 | 0.837 |
|  |  | ROS | 0.859 | 0.859 | 0.831 | 0.789 | 0.843 | 0.651 | 0.934 |
|  |  | SMOTE | 0.859 | 0.859 | 0.831 | 0.789 | 0.843 | 0.651 | 0.934 |
|  |  | ADASYN | 0.831 | 0.831 | 0.816 | 0.706 | 0.823 | 0.647 | 0.898 |
|  | Random | None | 0.831 | 0.831 | 0.791 | 0.790 | 0.807 | 0.615 | 0.854 |
|  |  | ROS | 0.873 | 0.873 | 0.850 | 0.858 | 0.858 | 0.683 | 0.846 |
|  |  | SMOTE | 0.775 | 0.775 | 0.811 | 0.621 | 0.787 | 0.750 | 0.845 |
|  |  | ADASYN | 0.845 | 0.845 | 0.845 | 0.751 | 0.844 | 0.773 | 0.890 |
|  | Hyperopt | None | 0.831 | 0.831 | 0.798 | 0.744 | 0.810 | 0.554 | 0.930 |
|  |  | ROS | 0.845 | 0.845 | 0.847 | 0.790 | 0.838 | 0.637 | 0.918 |
|  |  | SMOTE | 0.817 | 0.817 | 0.814 | 0.697 | 0.815 | 0.672 | 0.890 |
|  |  | ADASYN | 0.803 | 0.803 | 0.804 | 0.666 | 0.803 | 0.668 | 0.885 |

APPENDIX-IV. The appropriate values of hyperparameters of classifiers 1-5.

Classifier 1: C=44.5388, kernel = 'rbf', gamma = 0.1798.

Classifier 2: Criterion = 'entropy', maximum depth = 7, max features = 'sqrt', min samples leaf = 5, min samples split = 4, algorithm = 'SAMME', learning rate = 0.089, and number of estimators = 302.

Classifier 3: Criterion = 'gini', maximum depth = 3, max features = 'sqrt', min samples leaf = 1, min samples split = 2, algorithm = 'SAMME.R', learning rate = 0.0169, and number of estimators = 291.

Classifier 4: C=23.5253, kernel = 'rbf', gamma = 0.1269.

Classifier 5: Criterion = 'gini', maximum depth = 6, max features = None, min samples leaf = 9, min samples split = 2, algorithm = 'SAMME', learning rate = 0.1, and number of estimators = 350.

Classifier 6: Column subsample ratio = 0.3, gamma = 0, learning rate = 1, maximum depth =10, min child weight = 0, number of estimators = 300, and subsample ratio = 0.8.

Classifier 7: Column subsample ratio = 0.106, gamma = 0.0513, learning rate = 0.7915, maximum depth = 9, min child weight = 1, number of estimators = 246, and subsample ratio = 0.6619.
